# Using an evidence-based safety approach to develop China’s road safety strategies

**DOI:** 10.7189/jogh.09.020602

**Published:** 2019-12

**Authors:** Bing Wang, Chao Wu

**Affiliations:** 1 School of Resources and Safety Engineering, Central South University, Changsha, Hunan, PR China; 2 Safety & Security Theory Innovation and Promotion Center (STIPC), Central South University, Changsha, Hunan, PR China

## Abstract

**Background:**

Road accidents are a major global public safety and health problem. Presently, many countries such as China urgently need to find better strategies to improve their road safety. This paper has two key objectives, which are: (i) to propose potential solutions to improve China’s road safety, and (ii) to provide China and other countries with helpful evidence for their future road safety.

**Methods:**

This study attempts to use an evidence-based safety approach to propose some potential strategies for China’s road safety.

**Results:**

First, the current status of China’s road safety was analyzed. Second, major road safety problems in China were identified and discussed. Finally, this paper illustrates China’s road safety strategies based on available evidence.

**Conclusions:**

Presently and in the future, China’s road safety is facing a series of problems, such as increasing road safety management pressure, the weak road safety management foundation, and the lack of government supervision. To improve road safety, China should adopt a comprehensive strategy, which includes road safety risk prevention and control, road safety legislation, road safety supervision, road safety research and its application, road safety propaganda and education, and road safety culture, etc.

Road traffic not only provides great convenience for people’s life and travel but also plays a crucial role in regional economic and social development [1,2]. However, it has a tremendous impact on sustainable transportation, public safety and health because of the frequent occurrence of road accidents [3]. Road accidents are a worldwide public safety and health problem [4,5]. Therefore, in many countries, especially developing countries and major countries producing and using vehicles, such as China [2], road safety risks have raised major social concerns today. Moreover, China’s road safety still faces significant challenges with massive casualties and road accidents, as well as a high mortality rate [2,6-10]. Therefore, China has an urgent need to find better road safety strategies.

With the sustained and rapid development of China’s economy and society, and China’s rapid urbanization, motorization, and industrialization, there is a growing demand for road traffic [6,7,11]. As such, China’s road traffic has rapidly developed in recent years [7,11]. Unfortunately, with the rapid development of road traffic, road accidents have frequently occurred in China, representing a great threat to public safety and health, and creating a serious problem for road traffic development [7,8]. In China, a road accident is defined as an incident in which a vehicle causes (when on the road) personal injury, death, or property loss due to an error or unexpected incident [12]. Road accidents have resulted in numerous injuries and fatalities in China. They account for the largest percentage of all accidents and have become the first leading cause of death in various accidents [9]. Moreover, two crucial fatality indices, fatalities per 10^4^ motor vehicles and fatalities per 10^5^ licensed drivers in the road traffic in China are still much higher than those in developed countries such as the United States [7,8]. China’s road safety still faces substantial challenges with massive casualties and road accidents, as well as a high mortality rate. Additionally, in recent years (especially in 2016 and 2017), China has attached great importance to road safety and has proposed future targets for road safety in many significant policy documents, such as the *13th Five-Year (2016-2020) Plan for Road Safety* [8]. Therefore, it is of great urgency and importance for China to find better road safety strategies, to enhance future road safety, and to achieve a series of road safety objectives.

Although China’s road safety has been studied in some researhces [3,10], a complete solution to improve China’s road safety has not been developed. To propose potential solutions to improve China’s road safety, and to provide China and other countries with helpful evidence and references for their future road safety, this study proposes some potential strategies for China’s road safety.

## METHODS

This study attempts to use an evidence-based safety approach to propose some potential strategies for China’s road safety. In 2017, Wang et al. [13], inspired by evidence-based practice, developed a new and rigorous paradigm for safety decision-making, which is an evidence-based safety approach. To enhance the quality of safety decisions, this approach emphasizes that a safety decision should be based as much as possible on evidence (such as research findings), rather than on intuition, and unsystematic experience. Meanwhile, this approach is recognized as an excellent way to narrow the gap between safety research and safety practice [13]. The framework of this study can be designed according to the implementation steps of an evidence-based safety approach [13]. First, the present situation of China’s road safety was analyzed. Second, major road safety problems in China were identified and discussed. Finally, this paper develops China’s road safety strategies based on available research evidence. Moreover, this paper discusses China’s road safety at the national level.

The statistical data for this study comes from the National Bureau of Statistics of the People’s Republic of China (PRC), the Ministry of Emergency Management of the PRC, and the Blue Book of Road Safety in China 2015 [7].

## RESULTS

### The development of road traffic

**Figure 1:**
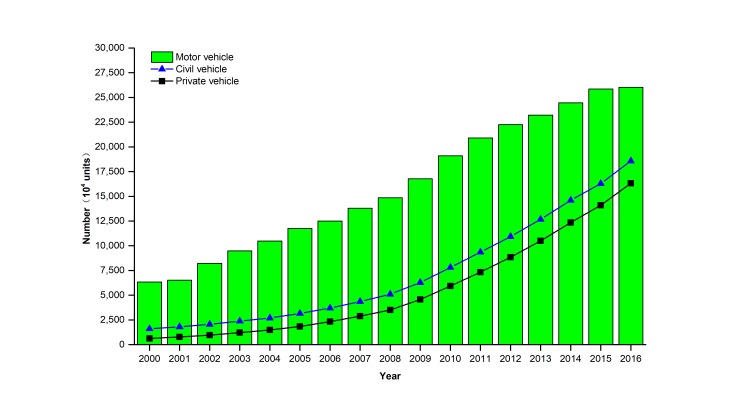
Numbers of motor vehicles, civil motor vehicles, and private vehicles in China from 2000 to 2016.

**Figure 2:**
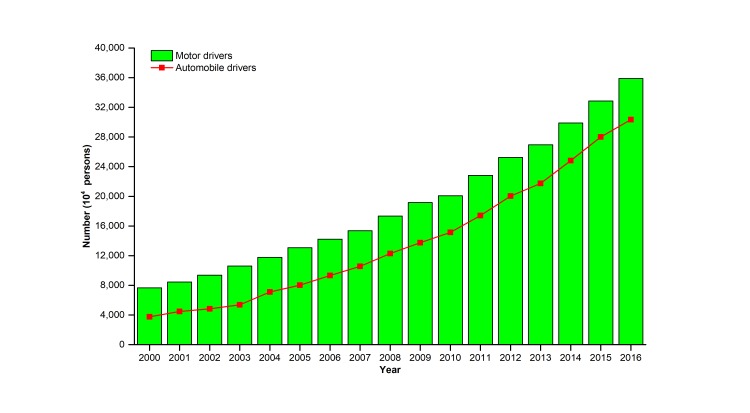
Numbers of motor drivers and automobile drivers in China from 2000 to 2016.

As the largest developing country, China has been experiencing rapid urbanization and motorization with the steady and rapid development of its society and economy. **Figure 1** shows the evolution of motor vehicles, civil motor vehicles, and private vehicles in China between 2000 and 2016. During this period, they all gradually rose. In 2016, the number of motor vehicles was 260 million, which was an increase of 410.94% compared to 2000. Meanwhile, in 2015, the number of electric bicycles exceeded 200 million [8]. Moreover, the numbers of both motor drivers and automobile drivers also significantly increased in 2000-2016, as shown in **Figure 2**.

Due to the rapid economic and social development, and the accelerated urbanization and motorization, the road transport industry in China has rapidly developed during the past decade, and a road network has steadily formed. **Table 1** demonstrates road transport investments and developments in China since 2005. In recent years, road transport investment in China is enormous and is increasing. For example, road transport investment reached 3293.73 billion Renminbi (RMB) in 2016 and accounted for 4.44% of the country’s Gross Domestic Product (GDP). In 2016, the total length of China’s highway reached approximately 4.70 million km, and the density of the road network in China reached close to approximately 48.9 km/100 km^2^, an increase of 40.39% and 143.3% compared to 2005. Meanwhile, China’s expressway mileage reached 131 000 km in 2016, an increase of 40.39% compared to 2005. According to the *13th Five-Year Plan for the Development of Modern Comprehensive Transportation System (2016-2020)* approved by the State Council of the PRC [11] in February 2017, the total mileage of highways and expressways in China will reach 5 million km and 150 000 km respectively by the end of 2020.

**Table 1:** Road transport investments and developments in China between 2005 and 2016

Year	GDP (10^9^ RMB)	Road transport investment (10^9^ RMB)	Road transport investment/GDP (%)	Highway mileage (10^4^ km)	Expressway mileage (10^4^ km)
2005	185 998.9	5581.4	3.00	334.52	4.10
2006	219 028.5	6481.6	2.96	345.70	4.53
2007	270 844.0	6926.6	2.56	358.37	5.39
2008	321 600.5	7411.5	2.30	373.02	6.03
2009	348 498.5	10 557.6	3.03	386.08	6.51
2010	411 265.2	12 764.5	3.10	400.82	7.41
2011	484 753.2	13 856.4	2.86	410.64	8.49
2012	539 116.5	17 466.4	3.24	423.75	9.62
2013	590 422.4	20 502.9	3.47	435.62	10.44
2014	644 791.1	24 513.2	3.80	446.39	11.19
2015	686 449.6	28 614.1	4.17	457.73	12.35
2016	741 140.4	32 937.3	4.44	469.63	13.10

GDP – gross domestic product, RMB – renminbi

**Table 2** shows the changes in passenger traffic, passenger-kilometers (passenger-km), freight traffic and freight ton-kilometers (ton-km) of highways in China between 1995 and 2012. During this period, due to the rapid development of road traffic, the amount of road transport has been increasing since 1995. Moreover, highways have been a dominant mode for transporting passengers. For example, the share of passenger-kilometers by highway reached 65.68% in 2015.

**Table 2:** Passenger traffic, passenger-kilometers, freight traffic, and freight ton-kilometers of highways in China between 1995 and 2015

Year	Passenger traffic (10^4^ persons)		Passenger-kilometers (100 million passenger-km)		Freight Traffic (10^4^ tons)		Freight ton-kilometers (100 million ton-km)	
**Total**	**Highways**	**Total**	**Highways**	**Total**	**Highways**	**Total**	**Highways**
1995	1 172 596	1 040 810	9002.0	4 603.0	1 234 810	940 387	35 730	4695
2000	1 478 573	1 347 392	13 155.1	7 207.1	1 401 177	1 056 312	47 591	6330
2005	1 847 018	1 697 381	17 466.7	9 292.1	1 862 066	1 341 778	80 258	8693
2010	3 269 508	3 052 738	27 894.3	15 020.8	3 241 807	2 448 052	141 837	43 390
2012	3 804 035	3 557010	33 383.1	18 467.5	4 100 436	3 188 475	173 804	59 535

### The current situation of road accidents

#### Road accidents, especially major road accidents are still frequent

**Figure 3:**
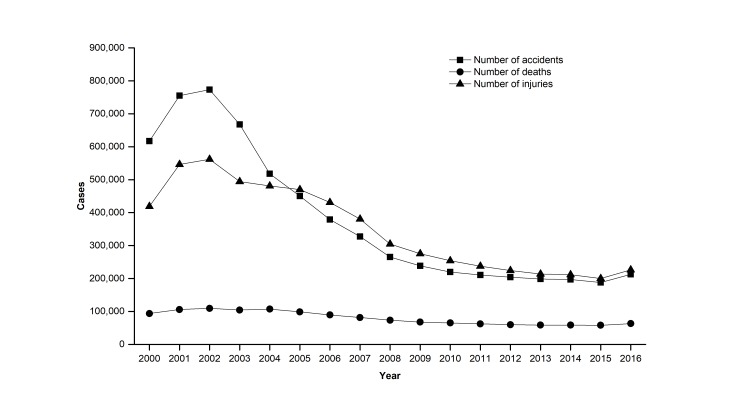
Numbers of road accidents, deaths and injuries in China from 2000 to 2016.

**Figure 4:**
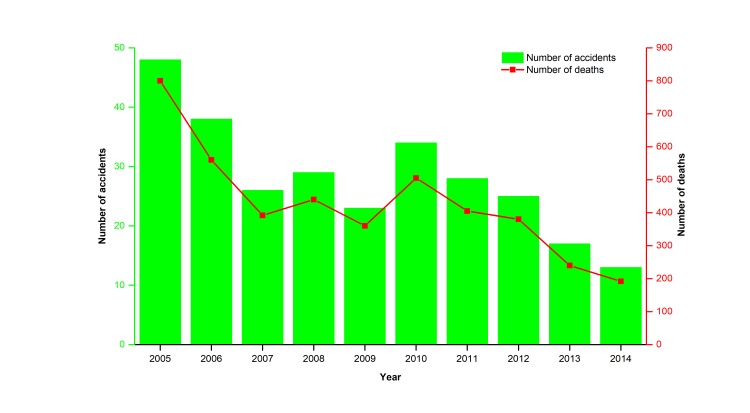
Numbers of road accidents each resulting in at least ten deaths, and deaths in China from 2005 to 2014. Source: Blue Book of Road Safety in China 2015 [7].

**Figure 5:**
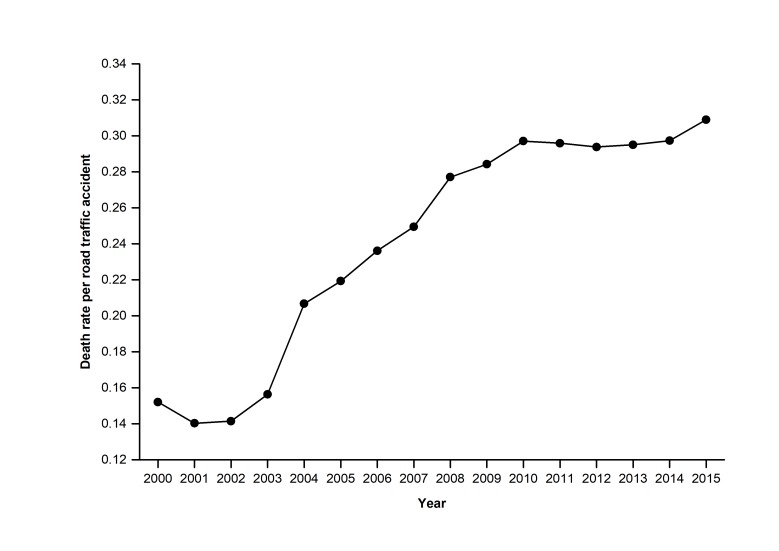
Death rate per road accident in China in each year 2000-2015.

The number of road accidents, the number of fatalities, and the number of injuries are three critical dimensions of the road safety record of a region. **Figure 3** shows the trends in the number of road accidents, fatalities, and injuries in China each year between 2000 and 2016. In this period, the number of road accidents and injuries rose before 2002 and then showed a steady decreasing trend. A primary reason for a dramatic decrease emerging in 2002-2003, possibly be that a special law on road safety, namely the *Road Safety Law of the PRC* [14] was approved and implemented in 2003. The number of fatalities in road accidents remained relatively unchanged. This trend shows that currently serious road accidents still happen frequently in China. The quantity variations of China’s road accidents each causing at least ten deaths and the corresponding death tolls from 2005 to 2014 are shown in **Figure 4**. Moreover, if we calculate an index of the death rate per road accident during 2000-2015 (see **Figure 5**), there has been an ongoing increase in the death rate per road accident in 2001-2015 apart from a quite slow decrease in 2010-2014, and this also shows that serious road accidents still frequently happen in China.

According to **Figure 3**, although the total number of road accidents and casualties in China has decreased in recent years, it is still substantial. For instance, presently, the number of fatalities in road accidents in China ranks second in the world [7]. Moreover, **Figures 3****,** **Figure 4****,** and **Figure 5** show that serious road accidents still occur frequently in China. Thus, despite considerable progress in some aspects, China’s road management still faces some significant challenges with substantial casualties and road accidents (especially serious road accidents).

#### Fatality indices are still high

**Figure 6:**
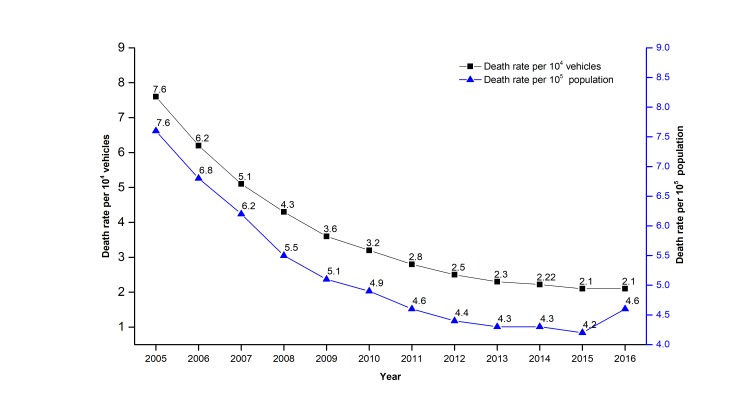
Death rate per 10^4^ vehicles and death rate per 10^5^ population in the road traffic in China from 2005 to 2016.

**Figure 7:**
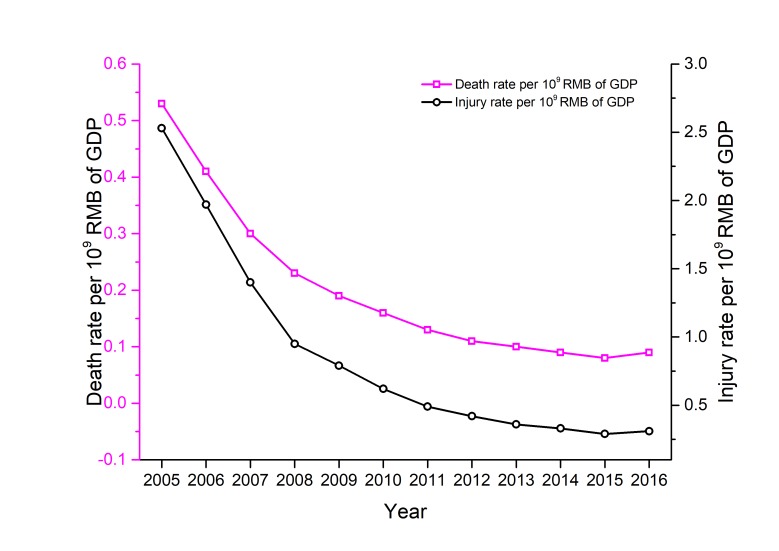
Death rate per 10^9^ RMB of GDP and injury rate per 10^9^ renminbi (RMB) of gross domestic product (GDP) in the road traffic in China from 2005 to 2016.

In most countries, such as the United States, Japan, and China, the death rate per 10^4^ vehicles and the death rate per 10^5^ population generally are two key indexes of road safety. **Figure 6** shows the evolution of the death rate per 10^4^ vehicles and the death rate per 10^5^ population in China between 2005 and 2016. According to **Figure 6**, these two indexes were declining year after year from 2005 to 2015. Meanwhile, according to the data of China’s GDP and casualties, this paper uses two calculated indexes, namely death rate per 10^9^ RMB of GDP and injury rate per 10^9^ RMB of GDP to explore the relationship between GDP and road accidents. With the increase of GDP, a decreasing trend of death rate per 10^9^ RMB of GDP, and of injury rate per 10^9^ RMB of GDP is noticeable (see **Figure 7**) during 2005-2015. This shows that China’s road safety situation has become relatively more stable in recent years. The steady decline from 2005 to 2015 needs a further explanation. Possible contributing factors mainly include: (1) the Chinese government’s off-road safety in road safety promotion (such as stricter road safety supervision and law enforcement), (2) continuous improvement of the legal system of road safety, (3) rapid improvement of the road traffic infrastructure, (4) rapid promotion of road traffic participants’ safety literacy, (5) effective control of risk factors related to vehicles, (6) wide application of road safety technologies and techniques, (7) nationwide road safety campaigns, and (8) rapid improvement of road safety services [7].

According to **Figure 6**, since 2011, the death rate per 10^4^ vehicles and the death rate per 10^5^ population slowly declined. Unfortunately, during 2015-2016, the death rate per 10^4^ vehicles was unchanged, and a moderate and substantial increase of the death rate per 10^5^ population emerged, as shown in **Figure 6**. The evolutions of death rate per 10^9^ RMB of GDP and injury rate per 10^9^ RMB of GDP are also similar to the evolution of death rate per 10^4^ vehicles and death rate per 10^5^ population, as shown in **Figure 7**. These results indicate that China is experiencing a bottleneck period for road safety promotion.

**Figure 8:**
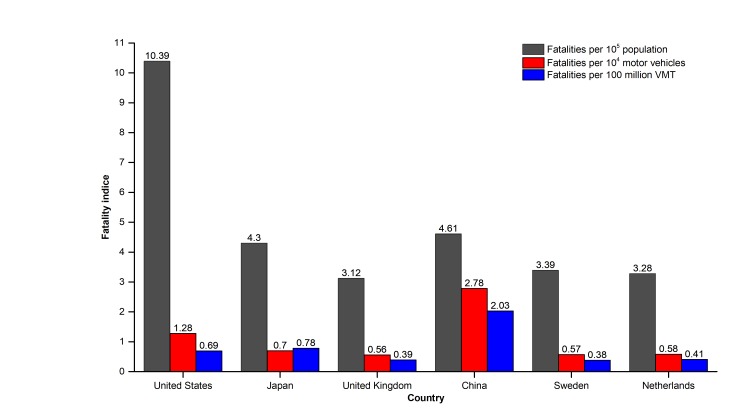
Comparison of some key fatality indices between China and some other countries in 2011. Source: Blue Book of Road Safety in China 2015 [7].

Furthermore, **Figure 8** indicates that, the number of deaths per 10^4^ motor vehicles, and deaths per 100 million Vehicle Miles of Travel (VMT) in China is 2-5 times higher than that of other developed countries (such as the United States, Japan, the United Kingdom, Sweden, and the Netherlands), and fatalities per 10^5^ population is also higher than Japan, the United Kingdom, Sweden, and the Netherlands. These results show that the road safety situation in China is still severe because of the high death rate. Why was China’s death rate from road traffic high? Two primary reasons are possibly the substantial road accidents and poor road safety emergency rescue.

#### The proportion of road accidents occurring on expressways increased

Although expressways can generally provide a good condition for road safety, many road accidents (especially major road accidents) occurred on expressways in the past decade mainly because of unsafe distances between vehicles, fatigue, and speeding [7]. For instance, the number of fatalities in road accidents each resulting in at least ten deaths and occurring on expressways was 1152 between 2005 and 2014 [7]. **Table 3** lists some of the representative road accidents that each resulted in at least 30 deaths and occurred on expressways in China from 2010 to 2014.

**Table 3:** Road accidents each resulting in at least 30 deaths and occurring on expressways in China between 2010 and 2014

Date	Location	Accident types	Vehicle types	Deaths
23/5/2010	Changchun-Shenzhen Expressway	Collision and fire	Semi-trailer truck and large sleeper bus	33
22/7/2011	Beijing-Hong Kong-Macao Expressway	Fire	Large sleeper bus	41
7/10/2011	Binbao Expressway	Scratch and rollover	Large bus	35
26/8/2012	Baotou-Maoming Expressway	Collision and fire	Large sleeper bus and small bus	36
1/3/2014	Jincheng-Jiyuan Expressway	Collision and fire	Methanol transport vehicle and coal transport vehicle	40
17/7/2014	Shanghai-Kunming Expressway	Collision and fire	Box/stake truck	54

Additionally, with the gradual increase of expressway mileage, the proportion of Road accidents occurring on expressways, and fatalities increased in recent years in China. For example, during 2011-2015, the road accidents occurring on expressways accounted for 4.4% of the total number of road accidents, and the fatalities of expressways accounted for 10.0% of the total fatalities of all road accidents. This was an increase of 0.4% and 1.7% respectively compared to during 2006-2010 [8]. Predictably, given the further development and increasing importance of China’s expressway, the proportion of road accidents occurring on expressways will possibly continue to increase in the future. China will need to propose some effective strategies and measures to prevent road accidents occurring on expressways through analyzing and studying their characteristics, tendencies, and causes in depth.

#### The current situation of road accidents occurring on rural roads is grave

In recent years, with the rapid improvement of rural road safety in China, the number of road accidents occurring on rural roads decreased. For instance, in 2014, the number of fatalities in road accidents occurring on rural roads was 14 787, and the number of road accidents was 46 359, which was a drop of 25.34% and 42.63% respectively compared to 2006 [7]. However, the current situation of road accidents occurring on rural roads in China is grave due to two main reasons, which are:

- Major road accidents occurring on rural roads are still frequent. For example, between 2010 and 2014, the number of fatalities in road accidents each resulting in at least ten deaths and occurring on rural roads was 427, and the number of road accidents was 33 (see **Table 4**). Among these road accidents, the highest percentage of road accidents is the off-road rollover accident (73.77%) [7]. Therefore, the prevention of off-road rollover accidents should be the focus of rural road safety management.

**Table 4:** Road accidents each resulting in at least ten deaths and occurring on rural roads in China between 2010 and 2014*

Year	Road accidents		Deaths	
**Number**	**Percentage**	**Number**	**Percentage**
2010	8	23.53	94	20.39
2011	8	29.63	118	25.93
2012	5	20.00	61	16.90
2013	7	43.75	99	47.60
2014	5	38.46	55	24.02
Total number	33	28.70	427	24.91

*Source: Blue Book of Road Safety in China 2015 [7].
- The proportion of road accidents occurring on rural roads, and fatalities is high and increased in recent years. For example, in 2014, the proportion of the road accidents occurring on rural roads and of the fatalities was 42.61% and 36.35% respectively [7]. During 2010-2015, an increasing trend of the proportion of fatalities in road accidents occurring on rural roads is evident, from 32.3% in 2010 to 37.2% in 2015 [8].

#### The proportion of road accidents occurring on urban roads increased

In recent years, the proportion of road accidents occurring on urban roads, and fatalities increased in China. For example, during 2011-2015, road accidents occurring on urban roads accounted for 37.2% of the total number of road accidents, and the fatalities of urban roads accounted for 24.3% of the total fatalities of all road accidents, which was an increase of 2.2% and 2.8% respectively compared to during 2006-2010 [8]. This trend shows that urban road safety management in China needs to be strengthened further.

#### The proportion of road accidents involving private vehicles rapidly increased

Along with the rapid increase of private vehicles (see **Figure 1**), the proportion of road accidents involving private vehicles and fatalities has rapidly increased in recent years. For instance, in 2015, the highest percentage of both road accidents and fatalities are the road accidents involving private vehicles (66.6% and 56.9%% respectively), an increase of 10.4% and 9.2% respectively compared to 2010 [8].

#### The number of road accidents involving electric bicycles rapidly increased

Because electric bicycles have many advantages such as simple operation, high speed, low price, low use-cost, and low environmental pollution, the number of electric bicycles has risen sharply in China’s urban and rural areas in recent years. By the end of 2014, there were 181 million electric bicycles in China. China has for years been the biggest electric bicycle market in the world [7]. With the surge in the number of electric bicycles, the number of road accidents involving electric bicycles and fatalities in China in each year was gradually increasing, and the proportion of road accidents involving electric bicycles, and fatalities has quickly increased in recent years. For example, the number of fatalities and injuries in road accidents involving electric bicycles in 2013 was respectively 8.14 and 6.07 times of that in 2004 [7]. In 2015, the road accidents involving electric bicycles accounted for 21.3% of the total number of road accidents, and the fatalities accounted for 13.7% of the total fatalities of all road accidents, which was an increase of 8.7% and 5.6% respectively compared to 2010 [8].

## DISCUSSION

### China’s road safety problems

According to the above analysis, as well as the *13th Five-Year Plan for Road Safety* [8], the *Blue Book of Road Safety in China 2015* [7], and other studies [2,6,9,10,15-19], presently, China’s road safety is facing eight major problems as follows.

#### Increasing road safety management pressure

The pressure of road safety management in China is gradually increasing with the rapid development of road traffic. By 2020, the overall GDP will exceed 90 trillion RMB, the per capita GDP will exceed 10 000 dollars, and the population urbanization rate will reach 60% [8]. In the road traffic industry, some changes will also occur [8], including: (1) the number of automobiles will increase from 172 million in 2015 to 250 million in 2020; (2) the number of cities with more than one million vehicles in China will increase from 31 in 2015 to 100 in 2020; (3) the number of motor drivers will increase from 327 million in 2015 to 420 million in 2020; and (4) the total mileage of highways and expressways in China will reach 5 million km and 150 000 km respectively by the end of 2020 [11]. Thus, the number of motor vehicles and motor drivers, and the amount of road transport in China will still proliferate, which must increase the difficulty, pressure, cost, and complexity of road safety management. Meanwhile, other problems affecting road safety such as urban traffic congestion may become more serious, which can pose higher requirements and more severe challenges for road safety management.

#### Problems of the road safety management system and mechanism

There are still some problems in the road safety management system and mechanism in China. Presently, although a basic system of road accident prevention and control has been formed, the road safety responsibility system and the collaboration mechanism among different departments (eg, the safety supervision department, the police department, the transportation department, the meteorological department, the quality inspection department, and the education department, etc.) need to be improved.

#### Problems of road traffic participants

There are two serious problems in the road safety propaganda and education for road traffic participants. First, although the road safety propaganda and education system have been primarily formed, there is still a lack of systematic road safety propaganda and education, and the safety consciousness and the civilized traffic concept of road traffic participants lag behind the rapid motorization and urbanization process. Second, the phenomenon of stressing driving skills and neglecting safety consciousness in driver training and examination was general, which is bad for developing the good driving habits (especially safe driving habits) of new drivers, as well as seriously impacting the education and training effectiveness of drivers.

#### Problems of vehicles

There are still many risk factors related to vehicles. First, the management of vehicle production and sales is lax. For example, the supervision for illegal enterprises producing vehicles lacks effective regulatory means. Second, the safety of vehicles for transporting dangerous cargo and large passenger vehicles should be further strengthened, and the safety of heavy-duty trucks and minibusses is poor. Third, the development of electric bicycles and low-speed electric vehicles (such as electric motorcycles and electric tricycles) is not regulated and guided effectively. Fourth, the vehicle driving safety assistant technology has not been widely applied in vehicle safety improvement. Finally, there is an urgent need to study and implement road safety policies for the development of new energy vehicles, intelligent connected vehicles, autonomous vehicles, and other new vehicle types.

#### Problems of the road

Road transport infrastructure construction cannot meet the needs of road safety. First, safety supervision for the plan, design, construction, and operation of roads needs to be further strengthened. Second, road safety facilities are poor and inadequate, and the “three simultaneousness” (designing the road safety facilities of a road construction project, constructing them, and using them for production and other business operations at the same time as the main body of the project) systems needs to be further implemented. Third, the standards for rural highway construction are low and work on the reconstruction or improvement of road tunnels, dangerous bridges, and accident-prone and high-risk roads is difficult. Fourth, the road safety investment of some local governments is insufficient, and road safety risk assessment is not effectively conducted.

#### Problems of road safety law-enforcement

There are still many defects in road safety law-enforcement in China, including: (i) the legal system for road safety is not perfect, and some road safety laws, regulations, and rules were unable to adapt to the current road safety situation and to meet the needs of current road safety law-enforcement; (ii) there are some serious problems (eg, undefined regulatory responsibilities, overlapping responsibilities, and lax supervision, etc.) in the safety supervision of vehicle production and sales, road transportation enterprises, as well as passenger and freight vehicles; and (iii) science and technology is not effectively applied to road safety law-enforcement.

#### Problems of road traffic emergency and rescue

Presently, road traffic emergency and rescue in China is relatively poor. The main problems existing in road traffic emergency and rescue in China are: (i) there are some problems in road traffic emergency management mechanisms (for example, China has not established a good road traffic emergency mechanism used by different regions, departments and industries, or a social mechanism for road traffic emergency rescue); (ii) the allocation of emergency resources is unreasonable; (iii) the emergency rescue technologies and equipment are outdated; (iv) the emergency workers lack professional knowledge and skills; (v) the capability to provide first aid for injured people in road accidents is still weak; and (vi) the secondary accidents caused by the poor emergency rescue or the lack of safety protection frequently occur.

#### Problems of scientific and technological support

Presently, road safety management in China still lacks effective scientific and technological support, such as: (i) research on the fundamental theories, key technologies, and advanced equipment of road safety is lacking, and cannot meet the needs of road safety management practices; (ii) the road safety-related information sharing mechanism among different departments is not well established; (iii) vast amounts of road safety-related data are not fully integrated, uncovered, or used at present; (iv) the standardized system for deep investigation and analysis of road accidents is not well established; and (v) the road accident investigation results are not used enough in road safety management practice.

### China’s road safety strategies made based on available evidence

Considering the above analysis, this section proposes some potential strategies for solving China’s road safety problems based on available evidence. According to modern road safety management thinking and approach, namely, systems thinking and approach (or “Safe Systems” models) [20-22], suggestions from some studies [6-8,15-19,23], and the road safety strategies of other countries [24-28], we suggest that China should adopt a comprehensive solution to improve future road safety. Specifically, this solution mainly includes seven strategies: (i) building a better road safety responsibility system; (ii) improving the road safety quality of road traffic participants; (iii) effectively controlling risk factors related to vehicles; (vi) effectively controlling risk factors related to roads; (v) improving road safety supervision and law enforcement; (vi) improving the emergency rescue capabilities for road accidents; and (vii) enhancing the role of science and technology in road safety.

#### Build a better road safety responsibility system

First, China should enhance the government’s road safety supervision responsibilities [7,23,29,30]. Specifically, four valid measures should be implemented: (i) strengthening the governmental leadership responsibility at all levels in road safety management to promote harmonious development between road safety, the economy, and society [9,29,31]; (ii) establishing and improving the rural road safety responsibility system, and implementing township government’s responsibility for rural road safety supervision; (iii) clearly defining the duties and responsibilities of road safety regulatory agencies [15]; and (iv) improving road safety supervision responsibility systems and specifying the road safety regulatory departments in some key areas such as the school bus safety and the road accident emergency rescue [19].

Additionally, China needs to strengthen the road safety responsibility of relevant enterprises (such as road transport enterprises, and enterprises engaged in motor vehicle production, sales, and modification) [23,29]. As such, six targeted strategies should be applied: (i) strengthening the safety evaluation of road transport enterprises; (ii) promoting the road transport enterprise’s safety standardization [20]; (iii) strengthening the safety risk prevention and control of road transport enterprises; (iv) establishing and applying a road transport enterprise’s credit system for road safety [9,32]; (v) strengthening the implementation of the relevant enterprise’s road safety responsibilities; and (vi) enhancing the road transport enterprise’s social responsibility to guide and promote enterprises to actively participate in road safety governance [8].

#### Improve the road safety quality of road traffic participants

China should first improve the road safety propaganda and education system. Accordingly, the following four key strategies will need to be developed and implemented: (i) formulating a public education program for road safety covering all stages of education (including preschool, compulsory, senior middle school, higher, and adult and elderly education) [8]; (ii) integrating road safety education to the national educational system [33]; (iii) enhancing the safety education and training of road transport enterprise’s employees [16]; and (iv) improving road safety propaganda and education through integrating social resources, implementing legal duties and responsibilities of the relevant departments (mainly including education departments and propaganda departments), and mobilizing the enthusiasm of news media, insurance enterprises, vehicle production enterprises, non-profit organizations, and other social organizations [16].

Second, China should develop effective road safety propaganda and education. For this, China will need to develop six strategies, which include: (i) taking road safety laws and regulations as an essential part of legal publicity and education [8]; (ii) developing road safety publicity and education activities in a series of social organizations (such as road transport enterprises, schools, communities, and cities, etc.), public areas, and rural areas [8]; (iii) implementing Internet-based road safety propaganda and education [16]; (iv) developing road safety propaganda and education activities themed around “safety belt is a lifebelt” to increase the use rate of safety belts [34]; (v) innovating technologies and approaches of road safety propaganda and education, such as developing scenario simulation methods, mobile tablet training [35,36], and interactive experience approaches; and (vi) formulating and implementing road safety propaganda and education programs and programs for key groups (such as motor vehicle drivers, primary and middle school students, urban migrant workers, tourists, and rural masses) through studying the characteristics of their road traffic behaviors.

Third, China should improve road safety awareness and driving skills of drivers [37]. To accomplish this, three measures will needed to be taken, which are: (i) improving the driving training examination system, and using training and education models of drivers to promote the cultivation of driver safety awareness and good driving habits [16]; (ii) making the qualification examination of motor vehicle drivers and its daily management stricter; and (iii) adding the emergency and first aid training in the driving training examination [8].

Fourth, China needs to establish a road safety credit system for road traffic participants [26]. Specifically, on the one hand, China needs to take the road safety-related information (such as traffic violations) of drivers into personal credit records to improve driver’s credit information management; on the other hand, China needs to establish a road safety announcement system.

Finally, China should develop a good road safety culture [36,38-40]. To achieve this goal, China should take the following seven key strategies according to the Production Safety Commission of the State Council (PSCSC) [8], which include: (i) building road safety culture parks; (ii) establishing a national road safety culture work production center; (iii) vigorously mobilizing social forces to make road safety culture programs and spread road safety culture; (iv) establishing a unique column concerning road safety culture in the mainstream media; (v) building a platform for broadcasting and downloading road safety culture information relying on the Internet; (vi) making full use of the convenience of the new media (eg, micro-blog, WeChat, mobile client, etc.) to spread road safety culture; and (vii) building a national road safety culture communication center by 2020.

#### Effectively control risk factors related to vehicles

First, China should strengthen the safety management of motor vehicles by using an integrated safety approach [41]. Accordingly, China will need to complete the following five tasks, which are: (i) improving safety standards for motor vehicles, especially vehicles transporting dangerous goods [42], trucks, large buses, and new energy vehicles; (ii) promoting the application of road safety technologies such as the autonomous collision avoidance system [43], the assisted driving system [44], the electronic stability program [45], and vehicle positioning [46]; (iii) strengthening the quality management of motor vehicles; (iv) promoting the legislation of vehicle production; and (v) improving the sharing mechanism for the information related to vehicle defects, accidents and injuries, and the defective vehicle product recall system [8].

Second, China should enhance the dynamic safety supervision of motor vehicles by fully implementing the *Dynamic Supervision and Management Measures for Road Transport Vehicles* [47]. According to PSCSC [8], there are some key tasks that need to be completed to achieve this objective, including: (i) strengthening safety supervision facing the life cycle of motor vehicles; (ii) strictly examining new vehicle registration; (iii) strengthening the inspection and supervision of the motor vehicle safety inspection; (iv) establishing a safety monitoring system for new energy vehicles [48]; (v) enhancing the regulation of overloaded vehicles and illegal truck modification; (vi) establishing a vehicle safety assessment mechanism to guide consumers to choose and buy safe vehicles; and (vii) studying the technical standards for intelligent connected vehicles, autonomous vehicles, and other new vehicle types.

Finally, China should strengthen the safety supervision of electric bicycles and low-speed electric vehicles [7,8]. For this, four effective strategies can be proposed according to PSCSC [8]: (i) revising the mandatory national standards for electric bicycles; (ii) strengthening the supervision for the production and sale of electric bicycles and low-speed electric vehicles; (iii) enhancing the management and policy guidance of the electric bicycle industry; and (iv) developing policies and measures for the disposal of in-use electric bicycles and low-speed electric vehicles failing to meet safety standards.

#### Effectively control risk factors related to roads

For effectively controlling risk factors related to roads, seven specific strategies can be suggested: (i) strengthening the implementation of road safety standards; (ii) developing a safety management system facing the life cycle of roads; (iii) strictly implementing the “three simultaneousness” systems; (iv) improving the safety of road infrastructure; (v) comprehensively implementing road safety assessment [49]; (vi) strengthening the construction of road safety facilities; and (vii) controlling risk factors related to rural roads [19].

#### Improve road safety supervision and law enforcement

First, China should improve the road safety law and regulation system from four aspects according to PSCSC [8], including: (i) evaluating the effectiveness of safety laws and regulations which have been implemented over five years; (ii) studying and revising the *Road Safety Law of the PRC*, the *Highway Law of the PRC*, the *Regulations on the Implementation of the Road Safety Law of the PRC*, the *Road Transport Regulations of the PRC*, the *Road Transport Regulations of the PRC*, the *Compulsory Insurance Regulations on Traffic Accident Liability for Motor Vehicles*, the *Regulations on School Bus Safety Management*, and so on; (iii) making serious violations of road safety laws and regulations (eg, “driving under influence,” “unlicensed driving,” “overloading,” etc.) included in the scope of the criminal law; and (iv) studying and developing road safety laws and regulations for intelligent connected vehicles, autonomous vehicles and other new vehicle types [7].

Second, China should improve the effectiveness of road safety supervision. Accordingly, four key strategies need to be developed and implemented: (i) enhancing the supervision on violations of road safety laws and regulations; (ii) implementing a road safety supervision system requiring different regions and departments to manage road safety to integrate road safety supervision resources jointly; (iii) strictly managing dangerous driving behaviors, such as dangerous driving behavior detection using video-extracted vehicle trajectory histograms [50]; and (iv) strictly investigating and affixing administrative accountability for major road accidents.

Finally, for enhancing road safety supervision and law enforcement capacities, China should take four specific strategies: (i) building a good road accident prevention and control system; (ii) using rural road safety management information systems in all rural areas [51]; (iii) strengthening the application of high-tech devices and information technologies in road safety supervision and law enforcement; and (iv) improving precise road safety management capacities.

#### Improve the emergency rescue capabilities for road accidents

First, China should improve the road accident emergency rescue mechanism [52]. As such, five measures need to be implemented: (i) establishing a road accident emergency rescue mechanism requiring different regions, departments, and industries to jointly participate in road accident emergency rescue [8]; (ii) building a basic information base for road accident emergency rescue [52]; (iii) establishing a sharing mechanism between the road accident data and the medical data, and a dynamic mechanism for the acquisition and transmission of road accident emergency rescue data; (iv) revising the *Emergency Response Plan of Road Traffic*, and improving the road traffic emergency plan systems of the country, the province, the city, and the county [8]; and (v) strengthening emergency rescue drills focusing on typical incidents (eg, adverse weather events, major road accidents, and road accidents involving dangerous goods, etc.) [7,9].

Second, China needs to adopt three strategies to increase financial support for the road accident emergency rescue: (i) improving the emergency material reserve system [53]; (ii) establishing a proper emergency material allocation and scheduling system [54]; and (iii) increasing investments on the study and application of emergency rescue equipment to improve the efficiency of emergency rescue.

Finally, according to PSCSC [8], Wang et al. [9] and Lv et al. [52], China should enhance the capacity-building of road accident rescue from seven aspects: (i) studying and formulating a guide to road accidents involving new energy vehicles; (ii) establishing good professional emergency rescue teams, and strengthening the cultivation of emergency rescue professionals; (iii) fostering emergency rescue service organizations, and encouraging volunteering organizations and insurance companies to participate in emergency rescue; (iv) developing an air emergency rescue mode; (v) establishing an expert consulting system for emergency rescue; (vi) improving the information base of emergency rescue and medical treatment; and (vii) improving the social assistance fund system for emergency rescue.

#### Enhance the role of science and technology in road safety

First, China should strengthen the basic theory and technology research regarding road safety. For this, three measures can be proposed according to PSCSC [8], Hughes et al. [22], the Research Institute of Highway Ministry of Transport and the China-Sweden Research Centre for Traffic Safety [7], and Wang et al. [55]: (i) developing innovative research on basic road accident prevention theories and technologies mainly aiming at driving behavior mechanisms and intervention, active and passive safety technologies of vehicles, safety technologies of new energy vehicles and autonomous vehicles, the road safety assessment and promotion, the road accident causation analysis, the evaluation of road accident losses, and so on; (ii) building the key state laboratory for road safety; and (iii) establishing the *National Key Research and Development Plan for Road Safety*.

Second, China should promote the application and sharing of scientific and technological achievements of road safety. Accordingly, three effective strategies can be suggested: (i) formulating and promulgating a special policy to guide and encourage the application of scientific and technological achievements of road safety; (ii) establishing a sharing mechanism and an application platform for scientific and technological achievements of road safety in the big data era; and (iii) implementing the road safety technology innovation guide engineering relying on research institutes, higher education institutions, enterprises, and other organizations.

Third, China should emphasize the application of road safety big data [56,57]. As such, four key strategies need to be taken [8]: (i) promoting the application and sharing of road safety-related information resources, such as sharing the national road accident database; (ii) establishing a dynamic system for road safety risk prediction and assessment, and a constant road accident analysis and forecast; (iii) applying big data, cloud computing and other information technologies to improve road accident analysis and forecast; and (iv) establishing a road safety-related information/data security assurance system to provide a strong foundation for the application of road safety information/data.

Finally, China should do an in-depth road accident investigation [58,59]. For this, three viable strategies [8,58,59] should be adopted: (i) strengthening and improving the collection of road accident data; (ii) innovating and improving approaches to cause analysis of road accidents; (iii) establishing a useful mechanism for road accident investigation; (iv) carrying out an in-depth investigation of major road accidents; and (v) according to the defects and problems found in the road accident investigation, revising and perfecting road safety standards and rules in time.

## CONCLUSIONS

China has always attached great importance to road safety. Since 2002, the number of road accidents and casualties has been steadily decreasing from a series of significant road safety measures and strategies adopted by the government. However, currently road accidents, especially major road accidents, still frequently occur in China, and fatality indices are still high. Moreover, the proportion of road accidents occurring on expressways and rural roads, and involving private vehicles and electric bicycles have increased in recent years. Thus, effective road safety management is urgently needed in China. Presently and in the future, China’s road safety is facing a series of problems, such as increasing road safety management pressure, the weak road safety management foundation, and the lack of government supervision. To improve road safety, China should adopt a comprehensive strategy, which includes road safety risk prevention and control, road safety legislation, road safety supervision, road safety research and its application, road safety propaganda and education, and road safety culture, etc. This paper can provide China and other countries with helpful evidence for their future road safety; and can promote the cooperation and exchange on road safety between China and other countries.
